# Supplementary material for: The opportunities and risks of mobile phones for refugees’ experience: A scoping review
Source: PLoS One. 2019 Dec 2;14(12):e0225684. doi: 10.1371/journal.pone.0225684 (PMC6886855; doi:10.1371/journal.pone.0225684)
Supplement: S1 Appendix — (DOCX) [file pone.0225684.s001.docx]

**S1 Appendix. Details of the 43 contributions reviewed.**

| **AUTHORS AND YEAR** | **CONTRIBUTION TYPE (RESEARCH DESIGN)** | **MIGRATION TYPE** | **TOPIC** |
| --- | --- | --- | --- |
| Alam, Imran (2015) | EMPIRICAL STUDY (Focus group) | 28 refugees (male and female) from diverse ethnic backgrounds living in Australia | MAINTENANCE AND DEVELOPMENT OF SOCIAL RELATIONS |
| Alencar, Kondova, Ribbens (2018) | EMPIRICAL STUDY (Interviews) | 16 males mainly from Syria living in Netherland | MEDIA PRACTICES |
| Bacishoga, Hooper, Johnston (2016) | EMPIRICAL STUDY (Ethnography) | 29 refugees living in South Africa | MAINTENANCE AND DEVELOPMENT OF SOCIAL RELATIONS |
| Beduschi (2018) | THEORETICAL ANALYSIS | Refugees journey | OPPORTUNITIES AND RISKS |
| Borkert, Fisher, Yafi (2018) | EMPIRICAL STUDY (Survey) | 83 Syrian refugees (male and female) living in Berlin | MEDIA PRACTICES |
| Bradley, Lindström, Hashemi (2017) | EMPIRICAL STUDY (Mixed methods) | 38 (males and females) newly-arrived Arabic speaking who granted asylum in Sweden and were involved in the national introduction program | EDUCATIONAL/THERAPEUTIC PURPOSES |
| Charmarkeh (2013) | EMPIRICAL STUDY (Ethnography) | 34 Somali refugees (male and female) living in three cities of France | MEDIA PRACTICES |
| Chouliaraki, Musarò (2017) | THEORETICAL ANALYSIS | Italy and Greece border sites of the 2015 migration “crisis” | OPPORTUNITIES AND RISKS |
| Curry, Croitoru, Crooks, Stefanidis (2019) | THEORETICAL ANALYSIS (with archive data) | Refugees journey | OPPORTUNITIES AND RISKS |
| Dahya, Dryden-Peterson (2017) | EMPIRICAL STUDY (Mixed methods) | 21 (interviews) Somali refugees + 248 (survey) Somali in diaspora | EDUCATIONAL/THERAPEUTIC PURPOSES |
| Dekker, Engbersen, Klaver, Vonk (2018) | EMPIRICAL STUDY (Interviews) | 54 Syrian (male and female) refugees | OPPORTUNITIES AND RISKS |
| Gillespie, Osseiran, Cheesman (2018) | EMPIRICAL STUDY (Ethnography) | 53 Syrian and Iraqi refugees | OPPORTUNITIES AND RISKS |
| Glazebrook (2004) | EMPIRICAL STUDY (Mixed methods) | 42 Hazara refugees (male and female) living in Australia | MEDIA PRACTICES |
| Harney (2013) | EMPIRICAL STUDY (Ethnography) | Three case studies of asylum seekers men living in Italy | MEDIA PRACTICES |
| Kang, Ling, Chib (2017) | EMPIRICAL STUDY (Interviews) | 20 North Korean refugees women living in South Korea | MAINTENANCE AND DEVELOPMENT OF SOCIAL RELATIONS |
| Kaufmann (2018) | EMPIRICAL STUDY (Mixed methods) | 10 Syrian refugees (male and female) living in Austria | MEDIA PRACTICES |
| Koh, Walker, Wollersheim, Liamputtong (2018) | EMPIRICAL STUDY (Mixed methods) | 111 Afghan, Burmese and Sudanese woman refugees living in Australia | MAINTENANCE AND DEVELOPMENT OF SOCIAL RELATIONS |
| Kutscher, Kreß (2016) | EMPIRICAL STUDY (Mixed methods) | 20 unaccompanied foreign minors living in young welfare institution in Germany | MAINTENANCE AND DEVELOPMENT OF SOCIAL RELATIONS |
| Kutscher, Kreß (2018) | EMPIRICAL STUDY (Mixed methods) | 20 unaccompanied foreign minors living in young welfare institution in Germany | MAINTENANCE AND DEVELOPMENT OF SOCIAL RELATIONS |
| Latonero, Kift (2018) | THEORETICAL ANALYSIS (with archive data) | Refugees journey | OPPORTUNITIES AND RISKS |
| Leurs (2014) | EMPIRICAL STUDY (Mixed methods) | 16 Somali young (male = 10 and female = 6) displaced in Ethiopia | MAINTENANCE AND DEVELOPMENT OF SOCIAL RELATIONS |
| Leurs (2017) | EMPIRICAL STUDY (Mixed methods) | 16 young refugees (males = 9 and females = 7) in Netherlands | SELF-ASSERTION AND SELF-EMPOWERMENT |
| Liamputtong, Koh, Wollersheim, Walker (2016) | EMPIRICAL STUDY (Mixed methods) | 111 Afghan, Burmese and Sudanese woman refugees living in Australia | MAINTENANCE AND DEVELOPMENT OF SOCIAL RELATIONS |
| Maitland, Xu (2015) | EMPIRICAL STUDY (Mixed methods) | 234 Syrian refugees living in Jordan camps | MEDIA PRACTICES |
| Mansour (2018) | EMPIRICAL STUDY (Mixed methods) | 37 Syrian refugees (male and female) displaced to Egypt | MEDIA PRACTICES |
| Mikal, Woodfield (2015) | EMPIRICAL STUDY (Focus group) | 12 Iraqi refugees and 13 Sudanese refugees (male and female) migrated in USA | MAINTENANCE AND DEVELOPMENT OF SOCIAL RELATIONS |
| Newell, Gomez, Guajardo (2016) | EMPIRICAL STUDY (Interviews) | 33 Mexican (male and female) who migrated to US | OPPORTUNITIES AND RISKS |
| O’Mara, Harris (2016) | THEORETICAL ANALYSIS (with participants' data) | 24 (males = 1 and females = 23) Vietnamese, Samoan, Sudanese, or Chinese young people in Melbourne with  migrant and refugee-background | EDUCATIONAL/THERAPEUTIC PURPOSES |
| Rae, Holman, Nethery (2018) | EMPIRICAL STUDY (Case study) | Analyses of two Facebook pages related to asylum seekers constrained from accessing Australia’s offshore immigration detention centres (the case of Behrouz Boochani, the case of ‘free the children NAURU’) | OPPORTUNITIES AND RISKS |
| Risam (2018) | EMPIRICAL STUDY (Text analysis) | Articles on migrant-related selfies in the context of the Syrian refugee crisis from 20 United States and United Kingdom newspapers. | OPPORTUNITIES AND RISKS |
| Rohde, Aal, Misaki, Randall, Weibert, Wulf (2016) | EMPIRICAL STUDY (Interviews) | Syrian FSA fighters, activists, refugees (males and females) (unknown number) | SELF-ASSERTION AND SELF-EMPOWERMENT |
| Sandoval, Torous, Keshavan (2017) | EMPIRICAL STUDY (Case study) | 1 male Eritrean refugee | EDUCATIONAL/THERAPEUTIC PURPOSES |
| Sijbrandij, Acarturk, Bird, Bryant, Burchert, Carswell et al. (2017) | THEORETICAL ANALYSES | Syrian refugees | EDUCATIONAL/THERAPEUTIC PURPOSES |
| Smets (2017) | EMPIRICAL STUDY (Ethnography) | 9 interviews with professionals; 60 hours of observations and 33 informal interviews with Syrian refugees living in a Turkish refugee camp | MEDIA PRACTICES |
| Sreenivasan, Bien-Aimé, Connolly-Ahern (2017) | EMPIRICAL STUDY (Interviews) | 12 Sri Lanka Tamil refugees residents of a camp in Indian | MAINTENANCE AND DEVELOPMENT OF SOCIAL RELATIONS |
| Tomita, Kandolo, Susser, Burns (2016) | EMPIRICAL STUDY (Survey) | 153 (baseline)/135 (follow-up) refugees in Durban (South Africa) | EDUCATIONAL/THERAPEUTIC PURPOSES |
| Tudsri, Hebbani (2015) | EMPIRICAL STUDY (Mixed methods) | 29 Hazara refugees young male living in Australia | MAINTENANCE AND DEVELOPMENT OF SOCIAL RELATIONS |
| Twigt (2018) | EMPIRICAL STUDY (Mixed methods) | 52 Iraqi refugees (males = 27 and females = 25) in Jordan | SELF-ASSERTION AND SELF-EMPOWERMENT |
| Veronis, Tabler, Ahmed (2018) | EMPIRICAL STUDY (Focus group) | 29 Syrian refugees young (male = 19 and female = 10) resettled in Canada | MAINTENANCE AND DEVELOPMENT OF SOCIAL RELATIONS |
| Walker, Koh, Wollersheim, Liamputtong (2015) | EMPIRICAL STUDY (Mixed methods) | 111 Afghan, Burmese and Sudanese woman refugees living in Australia | MAINTENANCE AND DEVELOPMENT OF SOCIAL RELATIONS |
| Wall, Campbell, Janbek (2017) | EMPIRICAL STUDY (Focus group) | 10 focus groups with Syrian (male and female) living in Jordan camps | MEDIA PRACTICES |
| Witteborn (2015) | EMPIRICAL STUDY (Mixed methods) | 176 asylum seekers (males and females) in Germany | SELF-ASSERTION AND SELF-EMPOWERMENT |
| Wollersheim, Koh, Walker, Liamputtong (2013) | EMPIRICAL STUDY (Focus group) | 9 Nuer (Sudan) refugees woman living in Australia | MAINTENANCE AND DEVELOPMENT OF SOCIAL RELATIONS |
