# Supplementary material for: The opportunities and risks of mobile phones for refugees’ experience: A scoping review
Source: PLoS One. 2019 Dec 2;14(12):e0225684. doi: 10.1371/journal.pone.0225684 (PMC6886855; doi:10.1371/journal.pone.0225684)
Supplement: Attachment — Submitted filename: RESPONSE TO EDITOR.docx [file pone.0225684.s003.docx]

COVER LETTER FOR - PONE-D-19-20003

Professor Tiziana Mancini

Department of Humanities, Social Sciences, and Cultural Industries
University of Parma, Italy

Professor Yann Benetreau

Associate Editor
*PLOS ONE*

September 2^th^, 2019

Dear Yann Benetreau,

I am writing for the article titled: ‘*The role of mobile phones in refugees’ experience. An integrative and critical review on the opportunities and risks for migrants’ human rights’* (**Original submission N. PONE-D-19-20003**).

My colleagues and I have discussed the feedback on our paper and we came to the conclusions that we wanted to resubmit our paper as a Scoping review as you suggested in your previous letter. We think that our paper re-titled “**The opportunities and risks of mobile phones for refugees’ human rights: A scoping review”** now better reflects the PONE high standard criteria for literature reviews, especially a Scoping review, which is the case of our manuscript.

Thus, building upon your suggestions and following the Scoping Review framework outlined by Arksey and O’Malley [2005], the recommendations by Levac et al. (2010), Munn et al. [2018], and Peters et al. [2017], as well as the example given by Pham et al. [2014], we carried out an extensive revision of the paper. More specifically, the following aspects have been included in the revised manuscript:

- Specification of the review’s aims and reasons for conducting a scoping review on the topic (see Introduction and 2.1 paragraph)
- Provision of details about the methodological framework (see Methodology section)
- Inclusion of specific information about the researchers involved with study selection and how disagreements were resolved (see 2.2 and 2.3 paragraphs). We also included the flowchart and uploaded the PRISMA-ScR CHECKLIST ITEM as supplementary material
- Reorganisation of Conclusion and Discussion through specific paragraph replying the aims of this scoping review
- Lastly, the manuscript has been entirely proofread by the London School of Economics and Political Sciences proofreading service.

I thank you very much for your kind attention.

Best regards,

Tiziana Mancini
