# Supplementary material for: The opportunities and risks of mobile phones for refugees’ experience: A scoping review
Source: PLoS One. 2019 Dec 2;14(12):e0225684. doi: 10.1371/journal.pone.0225684 (PMC6886855; doi:10.1371/journal.pone.0225684)
Supplement: Attachment — Submitted filename: Response to Reviewers.docx [file pone.0225684.s004.docx]

**Response to Reviewers**

We would really thank both you and the reviewers for the time spent in reviewing our manuscript PONE-D-19-24667, now titled “*“***The opportunities and risks of mobile phones for refugees’ experience: A scoping review***”*. We really believe that all the comments and suggestions have improved our paper and we hope that the new revision of the manuscript might be considered suitable for publication for PLOS-ONE.

We have addressed the reviewers’ comments point by point, and we have reported all the modifies suggested in the manuscript. Specifically, we have indicated in red the modified or added parts of the manuscript, and crossed out and highlighted in green the parts eliminated in order to shorted it, as requested by the first referee.

Sincerely,

Tiziana Mancini

**Reviewer #1:** This scoping review studies the literature on the role of mobile phones (MPs) in refugees’ experience in order to analyze the media practices in refugees’ everyday lives and if this use could be a risk or an opportunity for policies.
The manuscript is well organized ad well written. The authors present exhaustively the current state on this theme highlighting relevant aspects that already are known and gaps in the literature as well.
I think the methodology is well described and the discussion presents relevant points.
I have minor comments on content and methods (they follow), but I believe this review should be considered for publication.

- It is not clear why the systematic review flow (figure 1) presents authors in the search instead of the search enginges; moreover, PRISMA is mentioned but relevant methodological literature (Liberati, Moher, etc.) is not cited.

**AUTHORS REPLY**: As recommended by Levac et al. [2010], in this scoping review we used an interactive team approach to select and extract the research studies. This is the reason why we preferred to present authors rather than search engines in the search. However, we described search engines in details in the manuscript (see paragraph 2.2). With regard to figure 1, we changed the title as follows: “Flowchart of study selection process (adapted from the PRISMA statement by Moher and colleagues, 2009) and added the citation in the bibliography.

- Again on figure 1: the is something wrong in the numbers in the last phases ( 45 – 9 =37? …37+7=43? …probably the first one at full text screening is wrong); also, check the use of multiple character types in this figure (sometimes it is times new roman, sometimes calibri, etc.).

**AUTHORS REPLY**: Thank you very much for this advice: there was a mistake in the “Full text include in the review”: number id 36 and not 37! Multiple character types have been corrected.

- The main weakness of the manuscript is that it is quite long and complex. I think this is acceptable giving the richness of the topic, however some parts could be shortened, for example final discussion sometimes appears repetitive with results… maybe, it is not necessary to describe all the articles one by one, but just relevant examples, then commenting relevant aspects in discussion with frequent reference to the table in appendixes
Furthermore: some schematic concepts could be turn into figures, e.g., the “five topics along a continuum” – this would improve readability and citability of the manuscript and shorten it a little

**AUTHORS REPLY**: We reframed the discussion and conclusion taking your suggestion. Specifically, we tried to shortened it, being careful to synthetize and not just to repeat the results. Furthermore, we translated the idea of the continuum with polarities into a figure (Fig. 2).

- I would correct “conclusion and discussion” in “discussion and conclusion”

**AUTHORS REPLY**: Thank you. We have changed it accordingly.

- Last section could be shortened to focus on limitations (strengths have been made clear already in discussion in my opinion); moreover, authors sometimes merge limitations of the reviewed studies and limitations of their review itself, while only the latter should be mentioned here. In conclusion I think this section could be considerably shortened and just list possible methodological shortcomings and indications for future review efforts.

**AUTHORS REPLY**: Thank you very much for your suggestion. We renamed the paragraph 4.4 ‘Limitations and indications for future efforts’. Moreover, we briefly described the review’s strengths, focusing on the limitations of the review and not only on the studies reviewed.

**Reviewer #2:** I found this scoping review to be of a high standard. In particular: The study presents the results of original research, or, in this case, of a systematic and novel review of the literature. It is informative, and should meet the growing scholarly demand for a review of the literature on migration and on digital media, with a focus on mobile phones (MPs). The database and bibliographic searches were performed to a high logical and technical standard, and exhaustively so, and are described in sufficient detail. The article is presented in an intelligible fashion, without unnecessary jargon and technicalities, and is written in standard English. To the best of this reviewer's knowledge, this bibliography-based review research meets all applicable standards for the ethics of research integrity. Likewise, the article adheres to appropriate reporting guidelines and community standards for data availability.

**AUTHORS REPLY**: Thanks for appreciation!

The article acknowledges the limitations of the scoping review, which is important. On p. 34, line 828, the authors acknowledge as a limitation that "the predominantly qualitative nature of the studies analyzed does not allow generalization of the findings". I (this reviewer) find that this conclusion might be problematic. The authors themselves acknowledge, as one of their crucial areas of consensus on the basis of their lit review, that results are "ambivalent", and that ultimately MPs provide risks and opportunities. This is not a limitation, therefore: it is a finding. Likewise, the fact that much of the data is contextual (p. 34, line834) is inescapable. The very nature of these data does not allow them to be decontextualized, and it is the inclusion of "contexts" that makes these data meaningful in the first place.

**AUTHORS REPLY**: Thank you very much for this suggestion. We completely agree with this comment, especially with the fact that data considered could not be decontextualized. Coherently we deleted the reference to the qualitative nature of the data and of the different refugees’ ethnic background from the limitations of this review, focusing on two aspects: “First, it does not allow a comparison of the findings. Reasons for this can be found both in the objective circumstances of migration, i.e. factors related to the refugees’ migration experience [10], and in the different methodological aspects and contexts of the studies reviewed. However, it is important to emphasize that the nature of data included in this review does not allow them to be decontextualized; it is the inclusion of specific contexts that makes these data meaningful in the first place. Second, the limitation concern the use of human rights conceptual framework as a criterion for interpreting data and not also as a criterion to collect scientific contributions. This choice was related to the relatively limited dissemination of articles on this specific topic. Other studies must therefore be conducted to understand the role of mobile technologies in guaranteeing the application, enforceability and protection of asylum seekers and refugees fundamental human rights”.

One limitation is more serious, in my opinion, but it is not addressed: I do not believe "human rights" to be the correct conceptual framework for this article and the literature it surveys. I would actually recommend that "human rights" be excluded from the very title, and replaced with tropes that are also used in the article, such as affordances, refugee experiences, etc.
The excruciatingly vast literature on human rights is not discussed at all here -- this is legitimate, as the article already accomplishes a lot. But if so, "human rights" cannot be presented as a crucial focus, as the title would imply.
Human rights are briefly discussed on p4, lines 84-86; p15, lines 334-337; p12, lines 260-261; p.25, line 596. In this reviewer's opinion, this is neither systematic/exhaustive nor particularly original, and sometimes it reads as an afterthought.
In light of this, the research question posed by the authors (p5, lines 109-112) is ultimately a necessary and important one, save for the implications re: human rights, which are not really shown as to being "guaranteed", "applied", "enforced", or "protected" on the basis of this review of migration and digital media/MPs. I would urge the authors to consider amending the research question, by excluding "human rights" and including "opportunities and risks of MPs in refugees' experience" (p.9, line 200), e.g., "empowerment", "personal agency", "integration", access to "services" (p.33). I believe this revision (which I would still consider fundamental, as it is a conceptual/analytical one, albeit easily feasible) to the conceptualization of the article, and to some of its phrasing, would make it more compelling, by better aligning the data/literature, the authors' conclusions, and the expectations of PLOS readers.

**AUTHORS REPLY**: We substantially agree with the referee. Human rights literature is much wider than this manuscript might contain. An exhaustive and systematic literature on human rights was not the aim of this review and human rights did not mean to be the main conceptual framework for this article and for the literature we surveyed. If so, the expression “human rights” would have been included in the search query. Thus, as suggested, we changed the title in “The opportunities and risks of mobile phones for refugees’ experience” and the research question in “*Do mobile technologies, especially mobile phones, help refugees cope with the changes and the challenges of their migration experience, thereby promoting their security, empowerment, acculturation and wellbeing?”* in order to make the review more aligned with the search strategy and consequently with the data literature reviewed.

However, this scoping review started with the intention of using the results to reflect on the relationship between mobile technologies and human rights in refugees’ experience. Specifically, in line with JBI suggestions (e.g. Daudt et al., 2013) about the scoping review aims, one of our aims were “to inform practice and policymaking for advancing the use of MPs for the protection of migrants’ human rights, i.e. to propose recommendations for advancing the use of MPs for the protection of migrants’ human rights”. For this reason, in the **Discussion and conclusion** paragraph 4.2 we considered the opportunities and risks of mobile phones for refugees’ experience in terms of their opportunities and risks for the protection (or for the threat) of asylum seekers’ and refugees’ human rights. Human rights protection or threat were therefore used only as a criterion to discuss the empirical results of the review.

In order to make this clearer and not create in PLOS readers the expectation of reading a contribution on human rights, we better specified our third aim in both the abstract and introduction. Moreover, in the Discussion and conclusion paragraph, we changed every sentence or expressions that connected “human rights” to the research question, using human right just as a criterion to read and to discuss the empirical results of the review. We agree with the fact that this choice could be considered as a study limitation; therefore we specified this in paragraph 4.4.
